# Genetic/molecular alterations of meningiomas and the signaling pathways targeted

**DOI:** 10.18632/oncotarget.3870

**Published:** 2015-04-19

**Authors:** Patrícia Domingues, María González-Tablas, Álvaro Otero, Daniel Pascual, Laura Ruiz, David Miranda, Pablo Sousa, Jesús María Gonçalves, María Celeste Lopes, Alberto Orfao, María Dolores Tabernero

**Affiliations:** ^1^ Centre for Neurosciences and Cell Biology and Faculty of Pharmacy, University of Coimbra, Coimbra, Portugal; ^2^ Centre for Cancer Research (CIC-IBMCC, CSIC/USAL, IBSAL) and Department of Medicine, University of Salamanca, Salamanca, Spain; ^3^ Neurosurgery Service, University Hospital of Salamanca, Salamanca, Spain; ^4^ Instituto de Estudios de Ciencias de la salud de Castilla y León (IECSCYL-IBSAL) and Research Unit of the University Hospital of Salamanca, Salamanca, Spain

**Keywords:** genetic/molecular alteration, signal pathways, meningioma, chromosome 22

## Abstract

Meningiomas are usually considered to be benign central nervous system tumors; however, they show heterogenous clinical, histolopathological and cytogenetic features associated with a variable outcome. In recent years important advances have been achieved in the identification of the genetic/molecular alterations of meningiomas and the signaling pathways involved. Thus, monosomy 22, which is often associated with mutations of the *NF2* gene, has emerged as the most frequent alteration of meningiomas; in addition, several other genes (e.g. *AKT1, KLF4, TRAF7, SMO*) and chromosomes have been found to be recurrently altered often in association with more complex karyotypes and involvement of multiple signaling pathways. Here we review the current knowledge about the most relevant genes involved and the signaling pathways targeted by such alterations. In addition, we summarize those proposals that have been made so far for classification and prognostic stratification of meningiomas based on their genetic/genomic features.

## INTRODUCTION

Meningiomas are one of the most frequent primary brain neoplasias –30-35% of all cerebral nervous system (CNS) tumors–, which originate from the meningeal coverings of the brain and the spinal cord [1]. Despite most meningiomas correspond to histologically benign (i.e. WHO grade I) slow growing tumors [2], as a whole they display a broad spectrum of clinical, histological and cytogenetic features, even within the same WHO grade [3].

In recent years, important advances have been achieved in the identification and characterization of the genetic and molecular alterations of meningiomas and their association with the behavior of the disease. Consequently several chromosomal regions and candidate targeted genes have been identified. Monosomy 22/del(22q) in association or not with various mutations of the NF2 gene, is by far the most frequent cytogenetic event, potentially occurring at the early stages of the disease; however, other isolated chromosomal alterations and gene mutations, together with more complex karyotypes, have also been reported in meningiomas at relatively high frequencies, usually in association with a more aggressive tumor behavior [4].

Here we review the most relevant genetic and molecular alterations described so far in meningiomas, particularly focusing on the most frequently altered chromosomes, genes and signaling pathways.

### Genetic alterations of chromosome 22 in meningiomas

Monosomy 22 in association or not with mutation of the NF2 gene, is by far the most frequent chromosomal alteration in meningiomas although other genes in this chromosome, as well as other chromosomes have also been found to be recurrent altered in these tumors (Table 1).

**Table 1:** Most relevant genes involved in the pathogenesis of meningiomas and coded by those chromosomes more frequently altered in these tumors: chromosomal localization, type of genetic alteration and function

Gene	Locus	Product	Genetic alteration	Physiologic function	Role and/or impact on meningiomas
***Chromosome 22***					
*NF2*	22q12.2	Merlin	Downregulation Several mutations#	Linkage of cell membrane proteins to the cytoskeleton	Early event in tumorigenesis [1, 2] [3]
*BAM22*	22q12.2	Beta-adaptin	Downregulation	Endocytosis	Potential early event in tumorigenesis [4]
*BCR*	22q11	Bcr	Downregulation	Serine/threonine kinase, GTPase activator	Potentially involved in tumorigenesis [5]
*TIMP3*	22q12	Metalloproteinase inhibitor3	Hypermethylation	Inhibits MMP-2 and MMP9activity	Associated with high grade tumors [6]
***Chromosome 1***					
*ALPL*	1p36.1-p34	Alkaline phosphatase	Downregulation	Cell cycle control	Associated with high grade tumors and recurrence [7, 8]
***Chromosome 6***					
*HIST1H1C*	6p21.1	Histone H1.2	Upregulation	Cell cycle	Associated with recurrence [9]
*CTGF*	6q23.2	Connective tissue growth factor	Downregulation	Growth factor	Associated with recurrence [9]
***Chromosome 9***					
*CDKN2A/p16INKa*	9p21.3	P16	Downregulation; Hypermethylation	Cell cycle control	Associated with high grade tumors[10-13]
*CDKN2B/p15ARF*	9p21.3	P15	Downregulation; Hypermethylation	Cell cycle control	Associated with high grade tumors [10, 11, 13]
*CDKN2A/p14ARF*	9p21.3	P14	Downregulation; Hypermethylation	Cell cycle control	Associated with high grade tumors [10, 12-14]
*KLF4*	9q31	Kruppel-like factor 4	Upregulation K409Q mutation	Transcription factor which induces pluripotency	Associated with tumorigenesis of non-NF2 and secretory meningiomas [3, 15]
***Chromosome 14***					
*NDRG2*	14q11.2	NDRG2	Downregulation; Hypermethylation	Potentially involved in cell growth & apoptosis	Associated with high grade tumors and recurrence [16, 17]
*MEG3*	14q32	Noncoding RNA	Downregulation; Hypermethylation	Cell cycle	Linked to tumorigenesis & high grade tumors [18]
*AKT1*	14q32	Serine/threonine-protein kinase	Upregulation E17K mutation	Cell growth, Proliferation (activation PI3K pathway)	Associated with tumorigenesis of non-NF2 meningiomas [3, 19]
*TMEM30B*	14q	Transmembrane protein 30B	Downregulation	Cell cycle	Associated with tumor recurrence [9]
***Chromosome 17***					
*STAT3*	17q21.2	Signal transducer and activator of transcription 3	Upregulation	Transcription factor	Associated with high grade tumors [20, 21]
*RPS6K*	17q23	Ribosomal protein S6 kinase (p70^S6K^)	Upregulation	Cell growth, Proliferation	Potentially involved in tumorigenesis [22]
***Chromosome 18***					
*DAL-1*	18p11.32	4.1B	Downregulation	Links cell membrane proteins to cytoskeleton	Early event in tumorigenesis [23] / associated with progression [24]
*bcl-2*	18q21.33	Bcl-2	Upregulation	Regulator of apoptosis	Associated with high grade tumors and recurrence [25]
***Other chromosomes***					
*SMO*	7q32.3	Smoothened, G protein-coupled receptor	Upregulation Several mutations^$^	Cell growth, proliferation (activation Hh pathway)	Associated with tumorigenesis of non-NF2 meningiomas [3, 19]
*TSLC1*	11q23.2	CADM1	Downregulation	Cell adhesion	Associated with high grade tumors [26]
*TRAF7*	16p13.3	TNF receptor-associated factor 7	Several mutations*	Proapoptotic E3 ubiquitin ligase	Associated with tumorigenesis of non-NF2 meningiomas [3, 15]
*CDH1*	16q22.1	E-cadherin	Downregulation	Cell adhesion	Associated with high grade tumors, recurrence and invasion [27, 28]
*TIMP1*	Xp11.3-p11.23	Metalloproteinase inhibitor 1	Downregulation	Inhibits MMP-9 activity	Tumor invasion [29]

# Several NF2 mutations have been reported[30]. Only NF2 mutations reported by Clark et al[3] are listed here, underlined mutations were found in more than one tumor: K44X, W60X, Q115X, Y144X, Y153X, G161X, Y177X, Q178X, W191X, R198X, Y207X, L208P, Y217X, R262X, Q319X, Q324X, Q337X, R341X, E350X, Q362X, E366X, E427X, Q453X, Q459X, E460X.

$ SMO mutations[3, 19]: R113Q, L412F, L522V, W535L, P647S. The mutations in common in both studies were: L412F and W535L; underlined mutations were found in more than one tumor.

* TRAF 7 mutations reported by Clark et al[3] included T145M, F337S, C388Y, **G390R,** G390E, **T391I**, P398T, **N520S**, **G536S**, S561N, **K615E**, Y621C, Q637H, R641C, H642Q, H642P, R653P, **R653Q**, V665A; mutations reported by Reuss et al[15] were: Q384E, G390E, G390R, T391I, P398S, K498E, N520S, N520H, N520T, H521N, G536S, G559V, S561N, Y563C, Y577D, K615E, Y621N, R641L, R641H, R653Q. Mutations found in both studies included: G390R, T391I, N520S, G536S, K615E, R653Q and they are highlighted in bold; underlined mutations were found in more than one tumor.

### The NF2 gene and the merlin protein

A high proportion of meningiomas display recurrent genetic alterations of chromosome 22 and the *NF2* tumor suppressor gene coded in the long arm of chromosome 22 (22q). Accordingly, monosomy 22 is found in around half of meningioma cases, the great majority of NF2-associated meningiomas, as well as between 40-70% of sporadic meningiomas, displaying allelic losses (loss of heterozygosity, LOH) at the 22q12.2 chromosomal region, where the *NF2* gene is encoded [1, 3]. Additionally, up to 60% of these meningiomas carry inactivating mutations in the remainder *NF2* allele [1, 5], consistent with the classical two-hit hypothesis of tumor suppressor gene inactivation. So far, a range of *NF2* mutations have been reported in meningiomas most of which consist of small insertions, deletions, or nonsense mutations affecting the splicing sites [1, 6]. As the frequency of *NF2* mutation is roughly equal among the different WHO grades, it has been considered a relevant genetic alteration in tumor initiation rather than in malignant progression [1, 3]. Despite Lomas et al. [7] have reported that the *NF2* gene may be alternatively inactivated in meningiomas by aberrant promoter methylation, later studies indicated that methylation of the *NF2* promoter does not play a major role in meningioma development [8, 9].

The merlin protein (also known as schwannomin) is the product of the *NF2* gene. Merlin is a member of the 4.1 family of proteins which link integral membrane proteins to the cytoskeleton, and that are involved in the regulation of cell growth, proliferation and motility. Meningioma-associated *NF2* mutations commonly result in a truncated, non-functional protein, which may lead to abnormal cell growth and motility through destabilization of adherens junctions. The main characteristic of cells lacking the NF2 protein is the loss of contact-mediated inhibition of cell proliferation [5]. Additionally, loss of merlin activity has been associated with increased levels of ErbB receptors in primary Schwann cells, which regulate downstream mitogenic signaling pathways (e.g. Ras/Raf/MEK/ERK and PI3K/AKT); altogether, these findings support a relevant role of merlin in tumorigenesis in meningiomas [10]. In line with this hypothesis, mice which are heterozygous for *NF2* mutations more frequently develop metastatic tumors, and both *in vivo* and *in vitro* re-expression of wild type merlin leads to reduced tumor growth and decreased cell motility [3, 11].

Since the merlin functions include linking membrane proteins to the cytoskeleton, it has been hypothesized that alterations in merlin may substantially affect cell shape and might favor the appearance of a more mesenchymal-like phenotype rather than the epithelioid one, which is more commonly observed in wild type *NF2* meningiomas [1]. Of note, several studies have reported different frequencies of *NF2* mutation in meningiomas displaying distinct histopathological features; thus, alterations (e.g. monosomy) of chromosome 22q are more frequently observed in transitional and fibrous meningiomas than in the meningothelial variant [12, 13]. In addition, an association between the *NF2* gene and tumor localization has also been reported; Kros et al. [13] demonstrated that tumors of the convexity are more prone to have *NF2* mutations than anterior cranial-based tumors, and Clark et al. [14] recently correlated meningiomas with mutant *NF2* and/or chromosome 22 loss with tumor localization in the cerebral and cerebellar hemispheres. In line with the unique features associated with *NF2* mutation in meningiomas, an association with postmenopausal women tumors carrying monosomy 22 has also been reported recently [6].

### Other candidate genes coded in chromosome 22

Although *NF2* is the most frequently altered gene in chromosome 22, the frequency of deletions at this chromosomal region exceeds by far that of *NF2* mutations in meningioma, suggesting that other genes encoded at chromosome 22 may also be involved in meningioma tumorigenesis. In this regard, an early report found *BAM22* –a gene from the β-adaptin family coded at chromosome 22q12– to be inactivated in 9/71 meningiomas [15], and another more recent study found reduced expression of the *BCR* (breakpoint cluster region) gene coded at chromosome 22q11 in meningiomas with 22q LOH [16], further supporting the existence of candidate genes other than *NF2* in the pathogenesis of meningiomas (Table 1).

Tissue inhibitors of metalloproteinases (TIMP) are proteins that regulate matrix metalloproteinases (MMP) and thereby also cell proliferation, apoptosis, and angiogenesis. The *TIMP3* gene is coded at chromosome 22q12, is currently the best understood tumor suppressor gene being altered by epigenetic mechanisms in meningiomas [9]; of note epigenetic inactivation of TIMP3 has been recently associated with a more aggressive and higher-grade (grade II-III) meningioma phenotype [9, 17, 18].

### Other relevant chromosomes and genes in meningiomas

In addition to monosomy 22/del(22q), other isolated and combined chromosomal alterations have also been identified in meningiomas. Thus, losses of chromosomes 1p, 10, 14/14q, and less frequently also of chromosomes 6q, 9p, 18q and the sex chromosomes, together with gains of chromosomes 1q, 9q, 12q, 15q, 17q, and 20q, have all been recurrently detected in a variable proportion of cases that overall account for around 30% of all meningiomas [1, 4, 19-22]. Of note, several genes coded in these chromosome have been recently identified by next generation sequencing as recurrently altered in meningiomas; among others, these include the *AKT1* (E17K mutation), *SMO* (L412F and W535L mutations), *KLF4* (K409Q mutation) and *TRAF7* (several mutations mapped at the WD40 domains) genes [14, 23, 24]. Most interestingly, such mutations were shown to correlate with specific clinico-histopathological characteristics (e.g. tumor localization and histopathological subgroups) as well as with a subset of meningiomas lacking *NF2* mutation, bringing new insight into *NF2* non-mutated tumors [14, 23, 24].

Despite several other candidate genes have been proposed to be targeted by these chromosomal alterations, the specific relevant genes involved in many of them, still remain most frequently unknown.

### Genetic alterations of chromosome 1

Chromosome 1p deletions comprise the second most common chromosomal alteration in meningiomas [1, 3]. Del(1p) is typically associated with more aggressive meningiomas, its frequency increasing from grade I (13-26%) to grade II (40-76%) and grade III (70-100%) tumors [3, 25]; from the clinical point of view, loss of chromosome 1p is also associated with higher tumor recurrence rates [26, 27] (Table 1). The most frequently targeted regions of chromosome 1p involve the 1p33-34 and 1p36 cytobands [4], where several candidate genes have been identified, e.g. *TP73, CDKN2C*, *EPB41*, *GADD45A*, and *ALPL* [3]. However, current results do not support a key role in tumorigenesis and/or malignant progression for most of these genes as meningioma-specific tumor suppressors namely *CDKN2C, EPB41* and *GADD45A*, since they all failed to show consistent structural alterations. In contrast, although mutation of the *TP73* gene has not been found, methylation-mediated inactivation of *TP73* has been recurrently reported as frequent in meningiomas [17, 28]. Similarly, evidences exist about the potential role of the *ALPL* gene that encodes for the alkaline phosphatase enzyme at 1p36.1-34, as a tumor suppressor gene, because loss of chromosome 1p in meningiomas is strongly associated with loss of alkaline phosphatase activity [20], a predictor of meningioma recurrence [29]. Despite this, mutational analysis of *ALPL* has not been reported so far in the literature.

In turn, gains of chromosome 1q mainly involving two chromosomal regions (1q25.1 and 1q25.3 to 1q32.1), have been reported in around 60% of atypical meningiomas [30]. From the prognostic point of view, gains of chromosome 1q have been recurrently associated with a shorter progression-free survival [30]. However, detailed examination and identification of those genes with oncogenic potential which may be coded in this chromosome arm, still deserves further investigation.

### Genetic alterations of chromosome 6

Genetic losses involving the long arm of chromosome 6 are a relatively common finding in meningiomas, particularly among high grade tumors; reported frequencies range from 9% of grade I to 25-33% of grade II and 50-63% of grade III meningiomas [4, 21, 25] (Table 1). A common deleted segment at chromosome 6q includes the 6q24.1-qter region, where the *ESR1*, *IGF2R* [4], *DLL1*, and *CTGF* [31] cancer-associated genes are encoded. However, the functional role of these genes and the impact of their alterations in meningiomas are still far from being fully understood. In addition to del(6q), Pérez-Magán et al. [31] also reported overexpression of the histone cluster 1 genes coded at chromosome 6p (e.g. the *HIST1H1c* gene) in 27% and 89% of primary and recurrent meningiomas, respectively; recent results suggest that physical interaction of the H1.2 protein could be involved in epigenetic regulation of gene expression by maintaining specific DNA methylation patterns, but its functional role in meningiomas still remains to be elucidated.

### Genetic alterations of chromosome 9

Genetic losses at chromosome 9p have been reported in 5-17% of grade I, 18-52% of grade II, and 38-74% of grade III meningiomas [3] (Table 1). In contrast with other chromosomal alterations, the role of chromosome 9 in the development of malignant meningiomas is better defined, since it has been mostly associated with three tumor suppressor genes coded at chromosome 9p21: *CDKN2A/p16^INKa^*, *CDKN2A/p14^ARF^* and *CDKN2B/p15^ARF^*. Proteins coded by these three genes are all well known to play an important role in cell cycle regulation and apoptosis; thus p16^INKa^ and p15^ARF^ regulate cell cycle progression at the G1/S-phase checkpoint by inhibiting cyclin-cdk complexes, whereas p14^ARF^ regulates apoptosis through a blockade of MDM2-mediated degradation of p53 [3]. In addition, homozygous deletion and somatic mutation of these genes have been reported in anaplastic meningiomas, supporting the notion that inactivation of cell cycle regulation is an important feature of malignant progression [32]. For example, Boström et al. [33] found homozygous deletions of *CDKN2A/p16^INKa^*, *CDKN2B/p15^ARF^* and *CDKN2A/p14^ARF^* in 46% of anaplastic *vs.* 3% of atypical meningiomas. In a similar way, Goutagny et al. [34] have recently shown by SNP-arrays that the most frequent genomic alteration of meningiomas upon progression to grade III was loss of *CDKN2A*/*CDKN2B*. Additionally, inactivation through hypermethylation of CpG islands has also been reported in a smaller proportion of meningiomas, including hypermethylation of *CDKN2A/p16^INKa^* in 8-17% of cases, *CDKN2A/p14^ARF^* in 4-13%, and *CDKN2B/p15^ARF^* in 4% of these tumors [17, 35]. All such correlations also appear to have a prognostic impact since meningiomas with 9p21 losses have a significantly shorter survival and a worse clinical outcome than cases showing no alterations of chromosome 9p21 [36]. In addition to the *CDKN2A*/*CDKN2B* genes, the *KLF4* (Kruppel like factor 4) gene has also been recently reported to be mutated in meningiomas (particularly among secretory tumors) in association with lack of *NF2* mutation.

### Genetic alterations of chromosome 10

Losses of part or the whole chromosome 10 are present in a significant proportion of meningiomas, their frequency increasing from grade I (5-12%) to grade II (29-40%), and grade III (40-58%) tumors [3, 25, 37]. In addition, LOH at specific regions of chromosome 10 have been associated with both higher recurrence rates and a poorer survival [37]. Some of the potential candidate genes encoded in such chromosomal regions (e.g. 10q23-q25) include the *PTEN*, *MXI1* and *DMBT1* genes, but studies have failed to identify frequent/recurrent mutations of these genes in meningiomas [38].

More recently, Dobbins et al. [39] identified a new susceptibility locus for meningioma at chromosome 10p12.31, which encompasses the *MLLT10* (myeloid/lymphoid or mixed-lineage leukemia translocated to 10) gene; this gene is involved in chromatin remodeling and modulation of transcription. However, further investigations about the potential role of this gene in meningiomas are still needed.

### Genetic alterations of chromosome 14

Overall, monosomy 14/del(14q) represents the third most common chromosomal alteration in meningiomas, being present in up to 31% of grade I, 40–70% grade II, and up to 100% of grade III meningiomas [40]. From the prognostic point of view, the chromosome 14q status has been identified as a prognostic indicator for tumor recurrence [40, 41]. Because of this, among other chromosome 14 regions, the 14q32 region has been suggested to be potentially relevant for meningioma progression. In this regard, Zang et al. [42] have identified the *maternally expressed gene 3 (MEG3)* coded in this chromosomal region, as a candidate tumor suppressor gene with antiproliferative activity. *MEG3* is an imprinting gene that encodes for a non-coding RNA. In meningiomas, loss of *MEG3* expression, its deletion at the genomic DNA and the degree of methylation of its promoter, have all been associated with higher tumor growth [43] (Table 1). In turn, functional studies have demonstrated that MEG3 mediates its anti-tumoral effect through inhibition of DNA synthesis, colony formation and proliferation of meningioma cell lines, being also found to transactivate p53 [42]. Altogether, these findings suggest that MEG3 may have an important role as a novel long non-coding RNA tumor suppressor in meningiomas. Recently, the *AKT1* gene (coded at chromosome 14q32) has been reported to be mutated in meningiomas lacking *NF2* mutation [14, 23], the reported mutation (E17K) resulting in constitutive AKT1 activation [23]; therefore, the *AKT1* gene has become one of the most attractive target genes for cases with monosomy 14/del(14q).

Of note, the *N-myc downstream-regulated gene 2 (NDRG2)* has also been identified as a potential tumor suppressor gene coded at chromosome 14q11.2. Accordingly, *NDRG2* was found to be frequently downregulated at both the transcript and the protein levels in anaplastic meningiomas and in a subset of lower-grade and atypical cases with an aggressive clinical behavior [44], as well as in recurrent meningiomas [45]. Reduced expression of NDRG2 appears to be closely associated with hypermethylation of its promoter [44]. Despite the precise mechanism of action of *NDRG2* remains largely unknown, this gene has been involved in the regulation of cell growth, differentiation and apoptosis [9].

### Genetic alterations of chromosome 17

Chromosome 17 gains and/or amplification of the 17q21-qter chromosomal region have been recurrently reported, mostly in malignant meningiomas [4]. *RPS6K* (ribosomal protein S6 kinase; p70^S6K^) is a proto-oncogene coded at chromosome 17q23, which has been found to be overexpressed at the protein level [46]; however, amplification of this gene appears to occur only in a small subset of higher grade meningiomas, even when amplification of the loci adjacent to this gene is present [47], suggesting that other genes coded in the vicinity of *RPS6K* may be the main targets for 17q amplification. In this regard, recent studies have also investigated the potential role of *STAT3* (coded at 17q21.2), showing a higher frequency of enhanced expression of *STAT3* with increasing tumor grade [48] (Table 1). Zhang et al. [48] further reported that constitutively active STAT3 was significantly associated with expression of the vascular endothelial growth factor (VEGF), a major inducer of tumor angiogenesis, and Johnson et al. [49] suggested that the cerebrospinal fluid itself may act as a stimulus for STAT3 phosphorylation/activation.

### Genetic alterations of chromosome 18

Losses at chromosome 18q have been recurrently reported in meningiomas [3, 4, 50]; however, the specific targeted genes still remain to be identified. The expression of the bcl-2 oncoprotein coded at 18q21.3, has been associated with both a higher tumor grade and recurrence rate [51, 52]. In parallel, due to the role of the merlin protein in meningioma tumorigenesis, several studies have further investigated other members of the 4.1 family of membrane-associated proteins coded at chromosome 18q. These include the *DAL-1* (differentially expressed in adenocarcinoma of the lung) gene which encodes for the 4.1B protein and has been claimed to act as a potential tumor suppressor gene in meningiomas [53]. Thus, loss of heterozygosity of *DAL-1* at chromosome 18p11.32 was initially reported to occur in 60-76% of sporadic meningiomas [54], independently of the histological grade, suggesting that it could represent an early event in the pathogenesis of the disease (Table 1). However, more recent studies showed conflicting results. Thus, Yi et al. [55] reported that transgenic mice lacking *DAL-1* do not develop tumors, and Nunes et al. [56] reported that only 12/62 (19%) meningiomas had LOH of *DAL-1*, 11 of such 12 cases also showing LOH of the *NF2* gene. Altogether, these results suggest that *DAL-1* may be involved in progression rather than initiation of meningiomas, as supported by the presence of monosomy 18 and/or del(18p) in 3/4 WHO grade II tumors *vs.* 2/13 WHO grade I meningiomas [56]. Other recent reports in meningiomas found no losses involving the genomic regions which contain the *DAL-1* gene [8, 23], and another study found this gene to be mutated at a very low frequency [57].

### Altered signaling pathways in meningiomas

**Figure 1:**
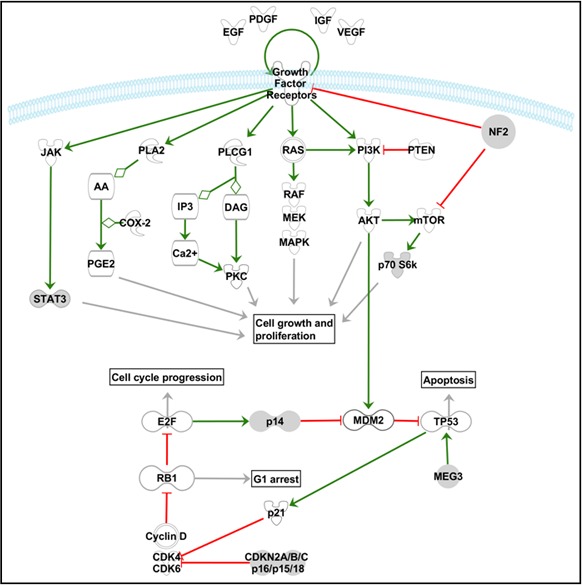
Schematic diagram illustrating the key elements of some of the most relevant signaling pathways involved in the pathogenesis of meningiomas The Ras-Raf-MEK-MAPK/ERK, PI3K-Akt/PKB, PLCγ1-PKC, PLA2-COX, JAK-STAT3 and mTOR signaling pathways are represented in the upper part of the scheme, while the relationships between the pRb and p53 cell cycle associated signaling pathways are illustrated in the lower part of the scheme. The pathway scheme displayed was generated with the Ingenuity Pathway Analysis (IPA) software (Ingenuity Systems Inc., Redwood City, CA, USA).

At present, it is well known that most of the above described genetic alterations have an impact on one or more signaling pathways which are recurrently involved in cancer. The most relevant and frequently altered signaling pathways in meningioma are reviewed in this next section and illustrated in Figure 1.

### The RB/p53 pathways and its impact on cell cycle dysregulation

RB has a central role in the inhibition of cell cycle progression at the G1/S-phase checkpoint. Briefly, RB binds (and inhibits) to the E2F transcription factor. Once cyclin D expression is upregulated (e.g. under mitogenic stimuli) it binds to either Cdk4 or Cdk6, and phosphorylates RB; RB phosphorylation induces release of the active E2F factor, leading to the transcription of genes which are critical for the transition from the G1 to the S-phase. *p16^INK4a^* and *p15^INK4b^* prevent S-phase entry by inhibiting the Cdk4/cyclin D complex [3]. In turn, the p53 pathway acts as a feedback inhibitor of the RB pathway, by inducing cell cycle arrest, DNA repair and apoptosis in case of aberrant RB pathway activation (Figure 1). The RB and p53 pathways are connected via p14^ARF^. Release of the E2F transcription factor following RB phosphorylation, also induces transcription of *p14^ARF^*, which promotes p53 activity through negative regulation of the MDM2 (murine double minute 2 protein) proto-oncogene. Dysregulation of these two pathways in higher-grade meningiomas is frequently associated with loss of p16^INK4a^, p15^INK4b^ and p14^ARF^, increased cell proliferation and tumor progression [33, 34]. In addition, accumulating evidences indicate that hypermethylation-associated loss of function of RB [8, 58], overexpression of the MDM2 gene/protein [25, 26] and loss of expression of *MEG3* (an anti-proliferative tumor suppressor that induces activation of p53 by a transcriptional effect) [42] in higher grade meningiomas, might further contribute to dysregulation of both cell cycle-associated pathways during meningioma progression.

### Growth factors and autocrine loops

Multiple studies have demonstrated enhanced expression of several growth factors, and activation of autocrine loops, which act as extra- and intracellular signals that induce tumor growth, cell migration, and angiogenesis, mostly via the MAPK and PI3K/Akt signaling pathways (Figure 1). Among others, the platelet-derived growth factor BB (PDGF-BB) and its PDGFR-β receptor have been found to be frequently overexpressed in meningiomas (typically at greater levels in high *vs.* low grade tumors), leading to meningioma cell proliferation via an autocrine and/or paracrine loop. Similarly, the epidermal growth factor receptor (EGFR), and both their EGF and transforming growth factor-alpha (TGFα) ligands, as well as some members of the insulin-like growth factor (IGF) system (e.g. IGF2) [59], have all been associated with meningioma cell proliferation and tumor progression. Interestingly, Lallemand et al. [10] reported that merlin regulates cell contact-mediated inhibition of proliferation by limiting the delivery of several growth factor receptors (e.g. ErbB2, ErbB3, IGF1R and PDGFR) at the plasma membrane of primary Schwann cells, and thereby inhibit the activity of the downstream mitogenic signaling pathways.

VEGFA and its VEGFR-1 receptor have been associated with regulation of the development of new blood vessels and peritumoral edema in brain tumors, a common feature in meningioma patients [3]. Of note, meningiomas express both VEGF and VEGFR, and VEGF expression correlates with the severity of peritumoral edema [60, 61] and tumor vascularization [61]. Despite this, the precise mechanisms that regulate VEGF expression in meningiomas remain unknown. In human cells, VEGF is mainly regulated by the hypoxia inducible factor-1 (HIF-1) transcription factor and in meningiomas, HIF-1 expression correlates with VEGF expression and the degree of peritumoral edema. In addition, upregulation of VEGF expression can also be induced by other growth factors such as EGF and PDGF, suggesting that both growth factors and hypoxia stimulation may all contribute to control VEGF expression in these tumors.

Other growth factors that have been associated with the pathogenesis of meningiomas include: 1) the stromal cell-derived factor 1 (SDF1) CXC chemokine and its CXCR4 receptor, which might exert its mitogenic effects through the MAPK pathway [62]; 2) the bone morphogenic proteins (BMPs) and their receptors (BMPR), which are associated with Smad 1 signaling, and; 3) the fibroblast growth factor (FGF) and its FGFR3 receptor, which are activated by the PI3K/Akt pathway [63]. In contrast, TGF-β and its receptors (TGF-βRI and TGF-βRII) may act as potential inhibitors of meningioma growth/proliferation through the Smad 2/3 apoptotic pathway [64], although the role of TGF-β in meningioma tumorigenesis remains to be fully established.

### The MAPK and PI3K/Akt signaling pathways

The mitogen-activated protein kinase (MAPK) pathway and the phosphatidylinositol 3-kinase (PI3K)/Akt pathway are both involved in multiple cellular processes (e.g. differentiation, growth, and apoptosis) associated with the pathogenesis of meningiomas, particularly with those tumors showing deregulated cell proliferation [3]. MAPKs are intracellular serine/threonine-specific protein kinases which are activated by extracellular stimuli (e.g., mitogen signals), leading to sequential activation of a kinase cascade triggered by the Ras/Raf-1/MEK-1/MAPK/ERK pathway and that ultimately, leads to phosphorylation/activation of transcription factors in the nucleus [64]. PI3Ks are a family of intracellular signal transducer enzymes that phosphorylate inositol phospholipids. Activation of PI3K results in phosphorylation/activation of PKB/Akt and subsequently p70^S6K^, which are key elements of the cell growth-promoting effects of this pathway; alternatively, activating mutations of AKT have also been recently reported in a subset of meningiomas [64]. In line with this, Johnson et al. [63] found evidences for the activation of both the MAPK and the Akt/PKB pathways in meningiomas, upon growth factor receptor signaling via e.g. PDGF-BB and PDGFβ; furthermore, these authors showed that administration of MAPK or PI3K inhibitors induces progressive growth inhibition of meningioma cells in association with reduced phosphorylation of MAPK or Akt and p70^S6K^, respectively. Interestingly, Mawrin et al. [65] have also found higher levels of phospho-Akt/PKB in association with lower levels of activation of MAPK in anaplastic and atypical *vs.* benign meningiomas; moreover, *in vitro* studies revealed decreased meningioma cell growth in the absence of apoptosis induced by a PI3K blocker, whereas inhibition of MAPK resulted in cell death through apoptosis [65]; based on these findings, the authors hypothesized that PI3K/Akt activation is associated with uncontrolled growth in malignant meningiomas, whereas MAPK activation appears to be involved in both meningioma cell proliferation and apoptosis [65].

### The PLCγ/PKC-calcium signaling pathway

Tyrosine kinase receptors also activate (e.g. phosphorylate) phospholipase C-γ1 (PLC-γ1), leading to hydrolysis of PIP2 (phosphatidylinositol 4,5-biphosphate) into two intracellular active second messengers: IP3 (inositol 1,4,5-triphosphate) and 1,2-DAG (1,2-diacylglycerol) (Figure 1). DAG activates protein kinase C (PKC), which enters the nucleus and activates transcription factors, resulting in cell proliferation and inhibition of apoptosis [64]. The activation of the EGFR kinase in meningioma cells further activates PLC-γ1 and increases its catalytic activity, leading to another mechanism that promotes meningioma cell growth; additional evidences indicate that PLCγ expression does not differ significantly between meningiomas of different histopathological grades [65]. In turn, IP3 mediates calcium release from intracellular stores resulting in increased free cytosolic calcium. Interestingly, calcium channel antagonists can block *in vitro* primary meningioma cell growth after stimulation with EGF and PDGF, as well as *in vivo* meningioma growth in a subcutaneous meningioma mouse model [66]; the specific mechanisms involved in such blockade of IP3-mediated intracellular calcium signaling pathways in meningiomas, still deserves further investigation.

### The cyclooxygenase-2 signaling pathway

The phospholipase A2 (PLA2)-cyclooxygenase (COX) signaling pathway has also been recently investigated in meningiomas [64]. COX-2 is an enzyme that serves as the rate-limiting step for the synthesis of prostaglandins from arachidonic acid. Prostaglandins (e.g. PGE2) are mediators of several critical cellular processes such as cell growth, proliferation, angiogenesis, suppression of apoptosis, and inflammation [3]. Normally, the cytoplasmic levels of arachidonic acid are relatively low, which limits the production of prostaglandins. However, altered levels of arachidonic acid and COX-2 overexpression have been associated with cancer growth and progression, possibly driven by signaling pathways such as those involving growth factors and the MAPK pathway [64]. Of note, high levels of arachidonic acid, increased prostaglandin production, as well as COX-2 overexpression [26, 67, 68], have all been reported in meningiomas. Moreover, COX-2 expression has been correlated with a greater degree of invasiveness to the brain or the adjacent soft tissues [67], tumor recurrence [26], a higher MIB-1 labeling index [68] and VEGF levels [67, 68], suggesting it may play an important role in the development and growth of meningiomas.

### The mTOR signaling pathway

Recent studies have found the mammalian target of rapamycin (mTOR) to be also involved in the signaling pathways associated with meningioma tumorigenesis [69, 70]. mTOR is a protein kinase that may be expressed in two distinct complexes (mTORC1 and mTORC2). mTORC1 regulates cell growth by promoting increased translation and protein synthesis through phosphorylation of the p70^S6K^ and 4EBP1 (eukaryotic translation initiation factor 4E-binding protein 1) effector proteins; in turn, mTORC2 directly phosphorylates Akt, a step required for its full activation [1, 3]. Recently, merlin has been identified as a negative regulator of mTORC1 and, James et al. [69] have demonstrated that mTORC1 levels are elevated in tumors derived from patients with *NF2* disease and in fibroblasts from an *NF2*-deficient mouse model. Thus, activation of mTORC1 has been associated with meningioma growth [69], and mTORC1 inhibitors have been shown to suppress meningioma growth in mouse models [70]. However, the exact mechanism through which merlin inhibits mTORC1 still remains unclear. In contrast to its effects on mTORC1, merlin positively regulates the kinase activity of mTORC2, downstream phosphorylation of mTORC2 substrates, including Akt, being reduced upon acute merlin deficiency in cells. Nevertheless, the attenuated mTORC2 signaling profiles reported in response to the loss of merlin, could not be detected in *NF2*-deficient meningiomas [69].

### The WNT/β-catenin pathway

The wingless (wnt)/β-catenin pathway has also been implicated in meningioma progression, through an altered expression of several of its genes. Thus, early studies based on microarray gene expression profiling identified increased expression of genes such as CTNNB1, CDK5R1, ENC1 and CCND1 [59]. Subsequently, Pecina-Slaus et al. reported LOH of the E-cadherin (*CDH1*) [71] and the adenomatous polyposis coli (*APC*) tumor suppressor genes in about one-third and half of the cases, respectively. Downregulation of E-cadherin expression in clinically aggressive and invasive meningiomas had already been described [72] in association with upregulation and nuclear/perinuclear localization of β-catenin [71], suggesting an important role for the WNT/β catenin pathway in meningioma tumorigenesis. Interestingly, Zhou et al. [73] suggested a model in which active merlin would inhibit Wnt/β-catenin signaling and maintain β-catenin and N-cadherin complexed at the plasma membrane; loss of merlin would then lead to loss of contact inhibition and activation of Wnt/β-catenin signaling, translocation of β-catenin to the nucleus and expression of intracellular growth-associated proteins such as c-myc and cyclin D1.

Interestingly, Pérez-Magán et al. [74] recently reported a gene expression profiling (GEP) signature of advanced and recurrent meningiomas, which included aberrant expression of genes of the Wnt pathway; thus, these authors found downregulation of *SFRP1*, a gene from the SFRPs (secreted frizzled-related proteins) protein family which are able to downregulate Wnt signaling, in recurrent and atypical meningiomas. Similarly, the *BCR* gene, which typically shows lower expression in meningiomas with LOH at chromosome 22q [16], has also been shown to act as a negative regulator of the Wnt pathway [75].

### The notch signaling pathway

The notch signaling pathway is involved in extracellular-to-intracellular signaling via the notch1-4 transmembrane proteins. Ligand proteins bind to the extracellular portion of the Notch proteins, resulting in proteolytic cleavage and release of the intracellular portion, which is translocated to the nucleus and initiates expression of the hairy/enhancer of split (HES) family of transcriptional regulators [3]. Cuevas et al. [76] comparatively analyzed the GEP of normal/reactive meninges and meningiomas of all histopathological grades, and demonstrated the potential involvement of the notch signaling pathway in meningiomas. Thus, HES1 expression was increased in all meningioma grades and it correlated with increased expression of notch1, notch2, and the jagged ligand [76]; in contrast, transducin-like enhancer of split (TLE) 2 and TLE3, two co-repressors that modulate HES1 activity, were specifically upregulated in malignant meningiomas [76]. Furthermore, deregulation of notch in meningiomas results in tetraploidy and chromosomal instability [77], further studies being required to elucidate the precise mechanism by which abnormal notch activation induces such genetic changes in meningiomas.

### The hedgehog (Hh) signaling pathway

When Hh binds its patched (PTCH) receptor, the smoothened (SMO) transmembrane protein is activated and initiates a signaling cascade that results in the activation of the GLI transcription factors (e.g. GLI1 and GLI2) and subsequent transcription of genes involved in cell growth, proliferation, angiogenesis, matrix remodeling, and stem cell homeostasis [3]. Recently, Laurendeau et al. [78] have analyzed the mRNA expression patterns of 32 Hh pathway-related genes in 36 meningiomas and found increased levels of 16 genes involved in the activation of the Hh pathway (e.g. *SMO*, *GLI1*, *GLI2*, *GLIS2*, *FOXM1, IGF2* and *SPP1)* and cell growth, together with decreased expression of 7 genes involved in the inhibition of the Hh pathway (e.g. the *PTCH1* tumor suppressor*)*; some of these genes further showed different expression profiles among tumors of different histopathological grades, suggesting distinctly altered profiles early during tumorigenesis *vs.* progression to more aggressive tumor lesions. Interestingly, recent reports have identified *SMO* mutations in meningiomas lacking *NF2* mutations [14, 23], which further supports the potentially relevant role of this pathway in the development of at least some meningiomas.

### Cytogenetic subgroups of meningiomas and their association with tumor progression

**Figure 2:**
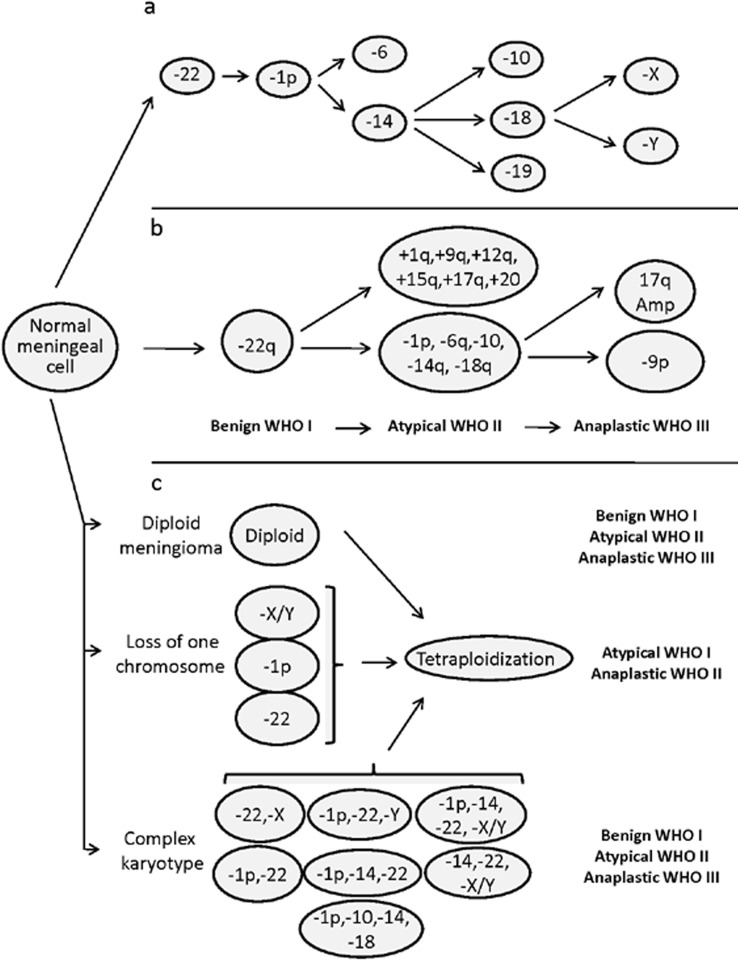
Patterns of cytogenetic alterations proposed to reflect cytogenetic progression of meningiomas, according to Ketter *et al* [79] (panel a), Weber et al. [25] (panel b), and Sayagues et al. [86] (panel c). Panel a, oncogenetic tree mixture model for the acquisition of chromosomal alterations in the development of meningiomas; the first two critical steps in the progression model correspond to monosomy 22, followed by loss of the short arm of chromosome 1. In the Weber et al model (panel b), progression from grade I to grade III is proposed to occur in parallel to the acquisition of specific chromosomal gains and losses at frequencies of more than 30% of cases; nevertheless, chromosomal changes may already have occurred in a lower grade in a smaller percentage of tumors. Finally, hypothetical intratumoral aneuploidization pathways defined on the basis of the patterns of clonal evolution observed for 11 chromosomes analyzed at the single cell level for individual tumors by Sayagues et al. (panel c).

Based on all what has been described above, at present it is well-established that meningiomas are cytogenetically heterogenous tumors. Consequently, for decades now, attempts have been made to classify meningiomas on cytogenetic grounds (Figure 2). The first cytogenetic classifications and cytogenetic models of progression of meningiomas were proposed in the 1990s [25]. Weber et al. [25] proposed a model of genomic alterations associated with meningioma progression based on comparative genomic hybridization (CGH) analysis of tumors of different grades (Figure 2). Later on, Ketter et al. [19] and Zang et al. [20], subdivided meningiomas into four subgroups based on their cytogenetic findings: Group 0, included meningiomas with a normal diploid chromosomal set; Group 1, consisted of tumors with monosomy 22 as the sole cytogenetic alteration; Group 2, was composed of tumors showing marked hypodiploidy with loss of additional autosomes, and finally; Group 3 included meningiomas with deletions of the short arm of chromosome 1, in association with other chromosomal aberrations such as loss of chromosome 22. Later on, these same authors applied oncogenetic tree mixtures to identify recurrent oncogenetic routes based on specific sequences of accumulation of somatic chromosomal changes in tumor cells (Figure 2); based on the routes identified, a genetic progression score (GPS) was built and used to categorize meningiomas into three groups of increasingly higher genetic complexity [79]. Subsequently, intratumoral cytogenetic patterns of clonal evolution have also been evaluated to establish recurrent pathways of cytogenetic progression at the single level, within individual tumors (Figure 2). Based on these later studies, complete or partial loss of either chromosome 22, a sex chromosome (i.e. chromosome Y in males and chromosome X in females), del(1p) or less frequently, monosomy 14/14q^−^ alone or in combination with other chromosomal losses (e.g., monosomy 10/10q^−^ and 18/18q^−^), would frequently represent the earliest detectable chromosomal alteration in meningioma cells. Interestingly, despite the clear association observed between a more advanced tumor grade and a higher number of tumor cell clones and complex karyotypes, authors found that the pathways of intratumoral clonal evolution observed in benign meningiomas were markedly different from those most frequently found in atypical/anaplastic tumors; altogether, these results suggest that the latter tumors might not always represent a more advanced stage of histologically benign meningiomas, but they could more likely correspond to stages of distinct clonal evolution pathways (Figure 2). For example, while monosomy 22 is commonly present in the earliest tumor cell clone observed in grade I meningiomas, it was only detected in a minor proportion of all atypical/anaplastic tumors; in contrast, presence of isolated losses of a sex chromosome, del(1p) and monosomy 14 in the ancestral tumor cell clone were characteristic of atypical/anaplastic tumors (Figure 2). In fact, although cytogenetic progression from low- to high-grade meningiomas may occur [22], it remains controversial [1] and the precise pathways of clonal evolution remain to be identified. This is due, at least in part, to the fact that data used to explain stepwise progression (e.g. cumulative acquisition of genetic alterations leading to more aggressive subclones), typically derives from cytogenetic analyses of different tumors of distinct grades, and from different patients. In contrast, studies addressing this question through the follow-up of patients showing tumor recurrence mostly reported no (or only minor) cytogenetic changes at recurrence, even after progression to a higher histopathological tumor grade [80].

From a clinical point of view, both the cytogenetic and genomic profile of meningiomas has recurrently been associated with the behavior of the disease [50, 80-86] (Table 2). Thus, both tumors carrying monosomy 22 and those displaying diploid karyotypes, have been associated with a better outcome [87] than that of cases with more complex karyotypes who show shorter recurrence-free survival rates [41, 88, 89]. Most interestingly, simultaneous presence of monosomy 14 and del (1p) in the ancestral tumor cell clones has been shown to be an adverse independent prognostic marker for disease-free survival in meningiomas [41].

**Table 2:** Genetic markers of meningiomas which have been associated with an independent prognostic value on patient relapse-free survival (RFS)

	Independent prognostic factors	Scoring criteria		Prognostic subgroups of meningioma patients	RFS rates at	
					5-years	10-years
Cai et al. 2001[31]	Tumor WHO grade -1p, -14			Grade II without -1p and-14q Grade II and -1p and -14q Grade III without -14q Grade III and -14q	98% 70% 35% 20%	80% 46% 35% 0%
Maíllo et al. 2003[32]	Age ≥ 45 vs < 45 years Tumor WHO grade −14	Score 0:	Age ≥ 45 years, grade I, no alteration of chr 14	Score 0 Score 1 Score ≥2	100% 82% 10%	100% 75% 0%
		Score 1:	Age < 45 years, grade II or grade III, alteration of chr 14			
Ketter R et al. 2007[33]	Accumulation of genetic events	Genetic progression tree score (GPS) <1.88 ≥1.88 and ≤6.39 ≥6.39		Group 0: GPS <1.88 Group 1: GPS ≥1.88 ≤6.39 Group 2: GPS ≥6.39	95% 88% 60%	88% 68% 0%
Jansen et al. 2012[34]	Age >55 or <55 years +1q32.1			Normal 1q32.1 and age <55 Normal 1q32.1 and age >55 +1q32.1 and age <55 +1q32.1 and age >55	88% 62% 53% 25%	60% 38% 42% 18%
Linsler et al. 2014[35]	−1p36			No del (1p36) del (1p36)	92% 65%	77% 22%
Domingues et al. 2014[36]	Age ≥55 vs <55 Tumor WHO grade Tumor size Tumor location Cytogenetic profile	Score 0:	Age ≥55 Grade I Diploid karyotype Tumor size <30mm Tumor localization other than cranial base (anterior) or intraventricular tumor	Low risk (score 0-1) Intermediate risk I (score 2-4) Intermediate risk II (score 5-6) High risk (score >7)	100% 93% 70% 50%	100% 85% 59% 0%
		Score 1:	Age<55 Grade II -22/del(22q) Tumor size: 30-50mm Cranial base(anterior) tumor			
		Score 2:	Grade III One altered chr other than chr22 Tumor size >50mm Intraventricular tumor			
		Score 3:	Complex karyotypes			

chr: chromosome

In parallel, single nucleotide polymorphism (SNP)-array studies have confirmed and extended the available information about the distinct cytogenetic subgroups of meningiomas [4, 21, 34]. Thus, Lee et al. [21] described 5 ‘classes’ of meningiomas based on gene expression analyses that showed a high correlation with their copy number alteration profile by SNP-arrays, as well as the tumor recurrence status and histopathology. Similarly, Tabernero et al. [4] confirmed by SNP-arrays that meningiomas can be classified into 3 major cytogenetic subgroups: diploid cases, meningiomas with a single chromosomal change [e.g. monosomy 22/22q^−^] and tumors with complex karyotypes including cases with ≥2 altered chromosomes. Of note, such cytogenetic classification of meningiomas was recently shown to be the most powerful independent predictor of outcome (e.g. 10–year recurrence-free survival rates of 91±6%, 90±4%, 67±8%, respectively) particularly when evaluated in combination with patient age and tumor size, localization and WHO grade [90].

Regarding GEP, Watson et al. [91], in a pioneering study in meningiomas, reported GEP of tumor cells to be associated with the WHO grade. Such association has been subsequently confirmed by others [59, 91, 92], and Fevre-Montange et al [92] and Serna et al [84] also reported an association of GEP with the main histopathological subtypes of grade I meningiomas. Similarly, unique GEP of meningioma cells have also been associated with tumor localization (e.g. spinal *vs.* intracranial tumors) [93], patient gender [94] and clinically relevant cytogenetic subgroups of meningiomas (i.e. diploid tumors vs cases with isolated monosomy 22/del(22q) vs meningiomas with complex karyotypes) [50].

Most interestingly, GEP of meningiomas have shown the existence of heterogeneous profiles which are associated with a different tumor behavior, even within the same WHO grade; thus, GEP of tumor cells from aggressive and/or invasive meningiomas emerged as being unique [44, 74, 84, 92] and typically associated with both high-proliferative gene expression signatures [95] and a high-risk of recurrence [50, 74, 84]. In this regard, Carvalho et al. [95] showed that meningiomas fall into two main molecular subgroups designated as ‘low-proliferative’ and ‘high-proliferative’ meningiomas, according to their different GEP and median MIB-1 labeling indices. Similarly, Perez-Magan et al. [74] identified a 49-gene signature of meningioma aggressiveness that characterizes histologically benign meningiomas which may recur.

## CONCLUDING REMARKS

Important advances have been achieved in the last decades in our understanding of the genetic/chromosomal alterations of meningiomas. Thus, current knowledge point out to the existence of several different pathways of tumorigenesis and clonal evolution which may target different genes in low vs high-grade tumors, as well as among low grade meningiomas. Thus, although *NF2* mutation in association with monosomy 22 is the most frequently observed alteration of a single gene, early mutations involving genes other than *NF2* (e.g. *AKT1, SMO, TRAF7, KLF4*) have recently emerged as alternative oncogenic pathways in NF2 non-mutated tumors. In addition, other genetic and/or chromosomal alterations are present in around one third of cases typically in the context of more complex karyotypes; such complex karyotypes appear to develop through multiple different pathways of clonal evolution which typically implicate several intracellular signaling pathways associated with cell proliferation, migration and apoptosis. Independently of the precise pathways of clonal evolution involved, accumulation of cytogenetic alterations, as reflected by more complex karyotypes with ≥2 altered chromosomes, usually leads to more aggressive disease, higher tumor grade and a poorer outcome in terms of recurrence-free survival. Consequently, assessment of tumor cytogenetics at diagnosis together with other clinical and histophatological features of the disease, becomes critical for both the establishment of optimal follow-up strategies and the definition of the most appropriate treatment for individual patients.
